# Synbiotic Kefir‐Yacon Drink Ameliorates Hypertension by Modulating Antioxidant and Anti‐Inflammatory Pathways

**DOI:** 10.1111/1750-3841.71598

**Published:** 2026-11-09

**Authors:** Flavia de Souza Andrade Moraes, Maressa de Carvalho Fragoso, Cristiane Lyrio da Silva, Karynna Alcantara Dutra, Lucas Gueller da Silva Gonçalves, Lucas Andrade Campelo, Antonio Ferreira de Melo Junior, Nazare Souza Bissoli, Girlandia Alexandre Brasil Amorim, Ewelyne Miranda de Lima, Tadeu Uggere de Andrade

**Affiliations:** ^1^ Department of Pharmacy University Vila Velha (UVV) Vila Velha Brazil; ^2^ iNOVA4Health, NOVA Medical School Universidade NOVA de Lisboa Lisbon Portugal; ^3^ Centro Clínico e Académico de Lisboa Lisbon Portugal; ^4^ Department of Physiological Sciences Federal University of Espírito Santo (UFES) Vitoria Brazil

**Keywords:** cardiovascular disease, oxidative stress, prebiotics, probiotics

## Abstract

Hypertension is associated with oxidative stress, chronic inflammation, and gut microbiota dysbiosis. This study evaluated whether a fermented kefir‐yacon synbiotic beverage could improve hemodynamic parameters in a hypertension model through its antioxidant, anti‐inflammatory, and gut microbiota‐modulating effects. Spontaneously hypertensive rats (SHR) and Wistar rats were divided into four groups: Wistar treated with vehicle (W), SHR treated with vehicle (SHR), SHR treated with unfermented yacon (SY), and SHR treated with the kefir‐yacon beverage (SKY). Treatments were administered orally for 8 weeks. Hemodynamic parameters, oxidative stress markers, transcription factors, antioxidant enzymes, pro‐inflammatory cytokines, cardiac hypertrophy, and selected fecal bacterial populations were assessed. The synbiotic beverage significantly reduced systolic, diastolic, and mean arterial pressure, restoring values comparable to those of normotensive animals (*p* < 0.05). Heart rate and cardiac hypertrophy were also reduced. In cardiac tissue, the beverage increased Nrf2 expression and antioxidant enzyme activities (SOD and CAT), while reducing lipid peroxidation (TBARS) and NF‐κB expression. Plasma TNF‐α and IL‐6 concentrations decreased (*p* < 0.05). Fecal counts of *Bifidobacterium* and *Lactobacillus* increased, indicating modulation of gut microbiota. The fermented kefir‐yacon beverage demonstrated antihypertensive, antioxidant, and anti‐inflammatory effects, suggesting its potential as a complementary nutritional strategy for managing hypertension.

## 1 Introduction

Hypertension remains a leading modifiable risk factor for cardiovascular diseases and a persistent global public health challenge (Yang et al. 2025). Increasing evidence has highlighted a close interplay between hypertension and the gut microbiota (Adamczak and Surma 2025). In particular, gut dysbiosis has been associated with reduced production of bioactive metabolites with antihypertensive properties, such as short‐chain fatty acids (SCFAs), alongside increased oxidative stress and chronic low‐grade inflammation—mechanisms that collectively contribute to the development and progression of hypertension (Abdolmaleky and Zhou 2024).

In this context, fermented foods have attracted considerable attention due to their capacity to modulate gut microbial composition and host physiology through multiple, complementary pathways (Gao et al. 2025). Probiotics, prebiotics, and synbiotics are recognized as effective strategies to beneficially alter the gut microbiota and promote host health (Swanson et al. 2020; Al‐Habsi et al. 2024). Notably, the antioxidant potential of synbiotics has been attributed to the combined or synergistic actions of probiotic microorganisms and prebiotic substrates, resulting in enhanced biological functionality (Mounir et al. 2022).

Recently, our group developed and characterized a non‐dairy synbiotic beverage based on yacon juice fermented with kefir grains. This formulation exhibited increased antioxidant capacity, elevated phenolic compound content, satisfactory microbiological viability, and no evidence of acute toxicity in vivo (Moraes et al. 2026). Although these findings support its safety and functional potential, its effects on cardiovascular dysfunction, particularly under hypertensive conditions, remain to be elucidated.

Kefir, a widely studied probiotic food, has been associated with improvements in metabolic, inflammatory, and cardiovascular parameters in both experimental and clinical settings (Beltrán‐Barrientos et al. 2018; Prado et al. 2015; Vieira et al. 2021; De Almeida Silva et al. 2020a; De Almeida et al. 2020b; Fijan et al. 2026). Additionally, studies investigating the combined use of yacon and kefir have reported beneficial effects on intestinal, immune, and metabolic outcomes, while also emphasizing the complexity of their underlying mechanisms (Gomes et al. 2023).

Given the central roles of oxidative stress, chronic inflammation, and gut microbiota alterations in the pathophysiology of arterial hypertension, synbiotic beverages combining prebiotic substrates and probiotic microorganisms represent a promising, yet insufficiently explored, nutritional approach. Therefore, the present study aimed to investigate the effects of a kefir‐yacon fermented synbiotic beverage on hemodynamic parameters, oxidative and inflammatory markers, and selected gut bacterial populations in spontaneously hypertensive rats (SHRs).

## 2 Materials and Methods

### 2.1 Preparation of the Fermented Synbiotic Beverage

The formulation and fermentation conditions of the synbiotic beverage were previously optimized and characterized by Moraes et al. (2026). Briefly, the selected formulation, containing 82.7% yacon juice and 2% kefir grains and fermented at 35°C for 24 h, was established using a Box‐Behnken experimental design based on antioxidant activity and pH. The selected beverage was subsequently characterized for proximate composition, pH, titratable acidity, microbiological composition, total phenolic and flavonoid contents, and antioxidant activity (ABTS and DPPH), as previously reported by Moraes et al. (2026).

The beverage used in the present study was prepared according to Moraes et al. (2026). Yacon roots were sliced and homogenized, followed by the addition of 0.1% (w/w) citric acid and processing in a food processor. Water was added to achieve the target formulation, followed by the incorporation of kefir grains. Fermentation was carried out for 24 h in a BOD incubator at 35°C. After fermentation, the beverage was filtered and refrigerated at 5°C for 24 h. A single batch was prepared for the entire experimental protocol, divided into aliquots, and stored at −80°C until use. Thus, all animals received beverage aliquots originating from the same production batch.

### 2.2 Ethical Considerations

The procedures were carried out respecting the ethical principles in animal experimentation and approved by the Ethics, Bioethics and Animal Welfare Committee of the Universidade Vila Velha (CEUA‐UVV, Opinion No. 509).

### 2.3 Experimental Animals

Eight‐week‐old male SHR and Wistar rats (Rattus norvegicus albinus), with an approximate weight of 250 g, were used. These animals were provided by the Experimental Monitoring Laboratory of the Biopractices Complex—UVV. They were housed in isolators within an Individually Ventilated Caging Rack ©Alesco, with humidity and temperature control, a 12‐hour light‐dark cycle, and ad libitum access to water and food (Probiotério feed, Moinho Primor, S.A.). The rats were randomly divided into 4 experimental groups (*n* = 10 for each group): male Wistar rats treated with vehicle (W); male SHRs treated with vehicle (SHR); male SHR treated with yacon (SY); and male SHR treated with the fermented beverage (Kefir + Yacon, SKY).

### 2.4 Treatment

Rats were treated daily for 8 weeks (0.3 mL/100 g body weight) orally (gavage) (Brasil et al. 2018). The animals in the Yacon + Kefir group received the Kefir + Yacon (KY) beverage with the synbiotic beverage most favorable antioxidant profile, as previously described (Moraes et al. 2026). Animals in the Yacon group received the unfermented (Y) beverage. Vehicle‐treated animals administered deionized water (W).

### 2.5 Experimental Protocol

#### 2.5.1 Evaluation of Hemodynamic Parameters

Systolic blood pressure (SBP), diastolic blood pressure (DBP), mean arterial pressure (MAP), and heart rate (HR) were measured by tail plethysmography (Insight, Ribeirão Preto, SP, Brazil), as previously described by Krege et al. (1995). Before the recorded measurements, the animals underwent daily acclimation sessions to the tail‐cuff procedure for 7 consecutive days to minimize stress associated with handling, restraint, and heating. For blood pressure assessment, the animals were heated in a bioheater chamber (Insight, Ribeirão Preto, SP, Brazil) to facilitate tail blood flow, followed by tail‐cuff measurements. Hemodynamic parameters were assessed before the beginning and at the end of the treatment period.

#### 2.5.2 Western Blot Analysis

The protein expression of the transcription factors Nrf2 and NF‐kB, as well as the antioxidant enzymes (superoxide dismutase—SOD and catalase—CAT), was analyzed in samples from the left ventricle (LV) of the experimental animals. The analysis of total LV proteins was performed using the Bradford method (Bradford 1976). The following antibodies were used: anti‐catalase (CAT, Sigma, USA), anti‐SOD (SOD; Sigma, USA), anti‐Nrf2 (Nrf2, Sigma, USA), and anti‐NF‐kB (NF‐kB, Sigma, USA).

The bands were visualized using a chromogenic substrate (NBT/BCIP Reagent Kit, Thermo Fisher Scientific, USA) and quantified using ImageJ software (National Institute of Health, NIH). The primary anti‐glyceraldehyde 3‐phosphate dehydrogenase (GAPDH) (1:3000, Santa Cruz Biotechnology, Santa Cruz, CA, USA) was used as an endogenous control, and protein expression levels were normalized to GAPDH expression (Nascimento et al. 2016). Details about the Western Blot analysis area provided in the Supplementary Material.

#### 2.5.3 Determination of Lipid Peroxidation (TBARS) and Advanced Oxidation Protein Products (AOPP)

Lipid peroxidation was analyzed by thiobarbituric acid reactive substances (TBARS), and protein oxidation by AOPP was performed on homogenized samples of cardiac tissue, according to Patočková et al. (2003) and Witko‐Sarsat et al. (1996), respectively. Protein quantification was performed by the Bradford assay (Bradford 1976).

#### 2.5.4 Determination of Pro‐Inflammatory Cytokines

Systemic (serum) levels of IL‐6 and TNF‐α were analyzed using the enzyme‐linked immunosorbent assay technique with kits from Invitrogen‐BioSource International (Camarillo, CA) in the blood of animals. The absorbance values were obtained from the readings performed using the Filter Max F5 Multi‐Mode Microplate Readers analyzer at a wavelength of 450 nm (Santanna et al. 2017).

#### 2.5.5 Cardiac Hypertrophy

After euthanasia, the ratio between the left ventricular mass (LVM) and body mass (BM) of the animals was used as an index of cardiac hypertrophy (Benova et al. 2013).

#### 2.5.6 Feces Collection and Microbiological Analysis

After euthanasia, feces were collected from the terminal intestine and immediately stored in sterile tubes for microbiological analysis (Sorokulova et al. 2016). Samples were serially diluted in sterile saline, then plated for colony counting and identification: *Bifidobacterium* sp. on bifidobacteria selective media (Sigma Aldrich) and *Lactobacillus* sp. on Man Rogosa and Sharpe agar (Sigma Aldrich). Plates were incubated at 37°C for 48–72 h under anaerobic conditions. Colony counts were performed on plates with 15–150 CFU, and results are expressed as log_10_ CFU/mL (Yu et al. 2019).

### 2.6 Statistical Analyses

The data were analyzed using one‐way ANOVA followed by Tukey's post hoc test. The analyses were performed using GraphPad Prism 8, and data are presented as mean ± standard error of the mean (S.E.M.); *p* < 0.05 was considered significant.

## 3 Results and Discussion

The present study demonstrates that a kefir–yacon fermented synbiotic beverage modulates key physiological and molecular pathways associated with hypertension, including oxidative stress, inflammation, and gut microbiota composition. These findings reinforce the concept that dietary interventions targeting the gut microbiota can exert systemic effects on host metabolism and cardiovascular function.

The principal finding is that the kefir–yacon beverage improves blood pressure control and heart rate in SHRs. To our knowledge, this is the first evidence showing that a beverage produced by fermenting a non‐dairy yacon matrix with kefir grains reduces blood pressure in this experimental model.

As expected, before treatment, SHR animals exhibited elevated SBP, DBP, MAP, and HR compared to Wistar rats (W) (Figure 1A,C,E,G). The kefir–yacon beverage significantly reduced SBP (W: 138.3 ± 4.18; SHR: 176.8 ± 3.47; SY: 170.8 ± 2.25; SKY: 155.5 ± 1.52 mmHg; *p* < 0.05) (Figure 1B), DBP (W: 108.3 ± 5.49; SHR: 141.5 ± 2.97; SY: 127.5 ± 2.10; SKY: 113.9 ± 1.46 mmHg; *p* < 0.05) (Figure 1D), and MAP (W: 123.3 ± 5.49; SHR: 155.3 ± 1.80; SY: 140.5 ± 1.5; SKY: 129.0 ± 1.61 mmHg; *p* < 0.05) (Figure 1F) compared to SHR and SY groups, with values not significantly different from W (*p* > 0.05). KY treatment also reduced HR (W: 274.5 ± 3.47; SHR: 397.1 ± 3.46; SY: 353.8 ± 5.32; SKY: 349.8 ± 3.67 bpm; *p* < 0.05) (Figure 1H) relative to SHR. Interestingly, the Y beverage alone also reduced DBP, MAP, and HR compared to SHR.

**FIGURE 1:**
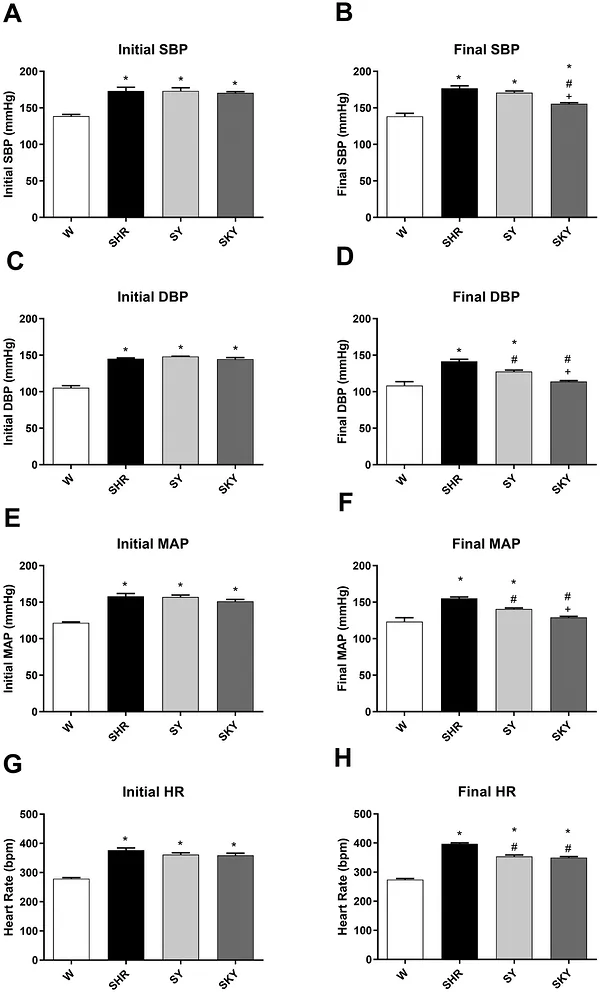
Results of blood pressure and heart rate measurements performed by means of plethysmography: (A) initial systolic blood pressure (initial SBP); (B) final systolic blood pressure (final SBP); (C) initial diastolic blood pressure (Initial DBP); (D) final‐diastolic blood pressure (final DBP); (E) initial mean arterial pressure (initial MAP); (F) final mean arterial pressure (final MAP); (G) initial heart rate (initial HR); (H) final heart rate (final HR). Data are expressed as the mean ± SEM values were considered significant for ^*^ *p* < 0.05 vs. W; ^#^ *p* < 0.05 vs. SHR; ^+^ *p* < 0.05 vs. SY.

Previous studies by our group (Friques et al. 2015; Brasil et al. 2018; Silva‐Cutini et al. 2019; De Almeida Silva et al. 2020a; De Andrade et al. 2021; Ghizi et al. 2021) and others (Kanbak et al. 2014; Bellikci‐Koyu et al. 2019; Chen et al. 2024) reported antihypertensive effects of milk kefir in experimental models (Kanbak et al. 2014; Friques et al. 2015; Brasil et al. 2018; Silva‐Cutini et al. 2019; De Almeida Silva et al. 2020a; De Andrade et al. 2021; Chen et al. 2024) and in humans (Bellikci‐Koyu et al. 2019; Ghizi et al. 2021). The present study extends these findings by demonstrating that yacon reduces blood pressure and HR in SHRs (SY group) and further improves these parameters when combined with kefir (SKY group), suggesting a synergistic effect of the synbiotic formulation.

The fermentation conditions used to produce the KY beverage increase antioxidant activity, phenolic compound content, and total counts of lactic acid bacteria and yeasts compared to the unfermented drink (Moraes et al. 2026). Based on these findings, we evaluated the antioxidant potential of the synbiotic beverage in cardiac tissue, observing increased expression of Nrf2 and the antioxidant enzymes SOD and CAT, which are essential for maintaining redox homeostasis.

Protein expression of total Nrf2 (Figure 2A) was increased in the SKY group compared to other groups (*p* < 0.05). KY treatment reduced total NF‐κB expression relative to SHR (*p* < 0.05), with no significant difference compared to normotensive animals (*p* > 0.05; Figure 2B). SHR animals showed reduced total SOD compared to W (*p* < 0.05) (Figure 2C). KY normalized total SOD expression in cardiac tissue, with levels higher than SHR and SY (*p* < 0.05) and not different from W. KY also increased total CAT expression in heart tissue compared to hypertensive, W, and SY groups (*p* < 0.05). The Y beverage alone increased total CAT compared to W and SHR (*p* < 0.05).

**FIGURE 2:**
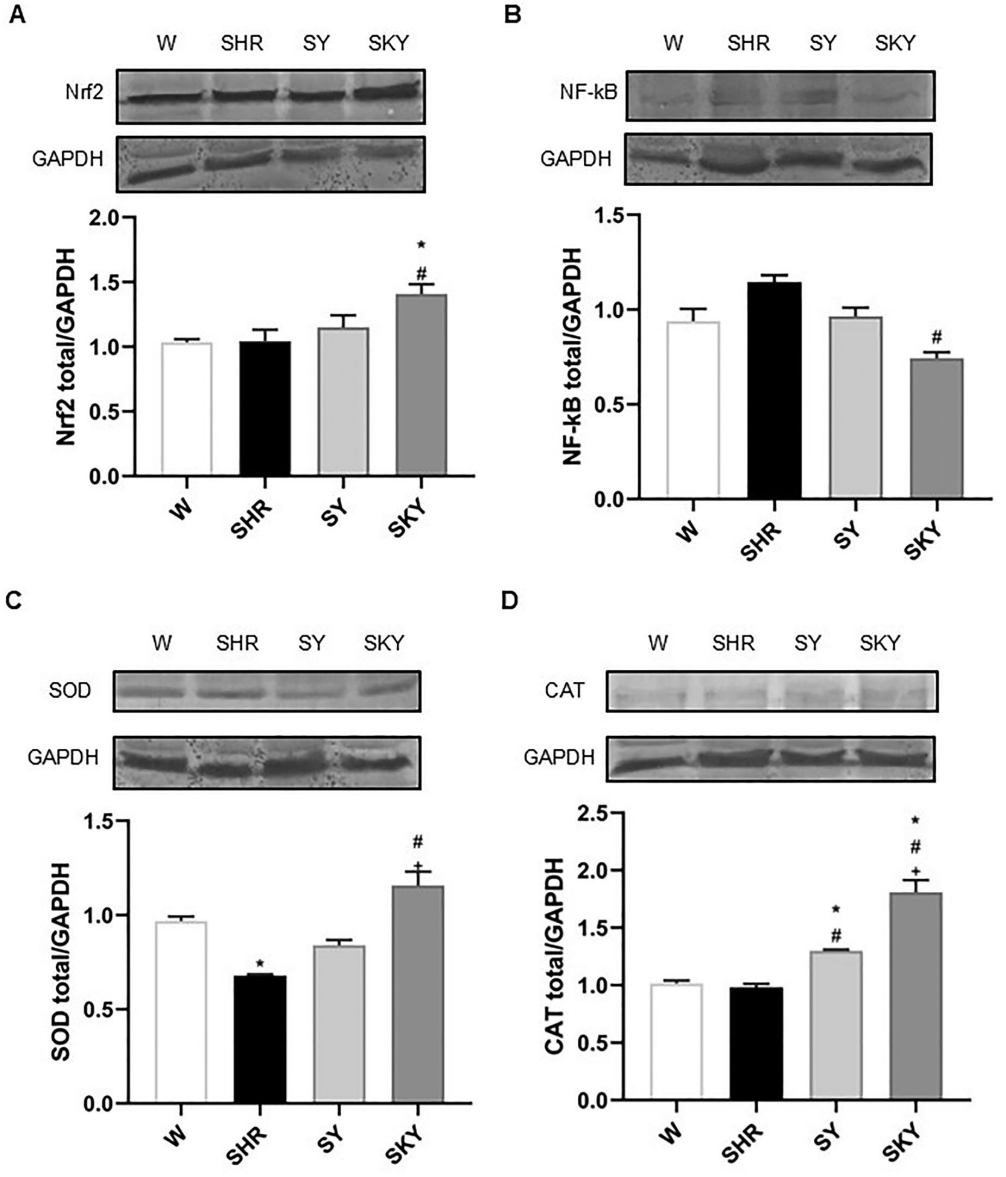
Protein expression of (A) Nrf2, (B) NF‐kB, (C) SOD, and (D) CAT in the cardiac tissue of experimental animals. Data are expressed as the mean ± S.E.M. The values were considered significant for *p < 0.05 vs. W; ^#^ *p* < 0.05 vs. SHR; ^+^ *p* < 0.05 vs. SY.

No significant differences in AOPP were observed among groups (*p* > 0.05; Figure 3A). SKY animals showed normalization of TBARS values in cardiac tissue, with values not significantly different from W (*p* > 0.05) and reduced compared to SHR and SY (*p* < 0.05) (Figure 3B). KY treatment reduced plasma levels of IL‐6 (Figure 4A) and TNF‐α (Figure 4B) compared to hypertensive animals (*p* < 0.05).

**FIGURE 3:**
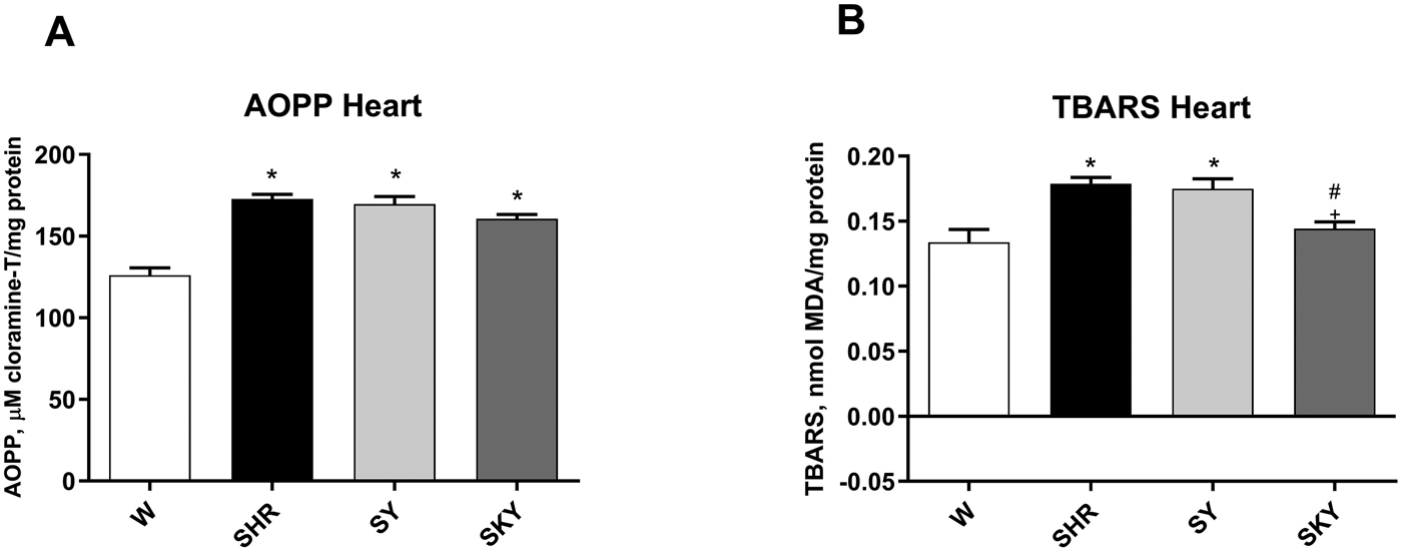
Results of oxidative stress markers in cardiac tissue. (A) Evaluation of advanced oxidation of protein products (AOPP); (B) thiobarbituric acid reactive substances (TBARS). Data are expressed as the mean ± S.E.M. The values were considered significant for ^*^ *p* < 0.05 vs. W; ^#^ *p* < 0.05 vs. SHR; ^+^ *p* < 0.05 vs. SY.

**FIGURE 4:**
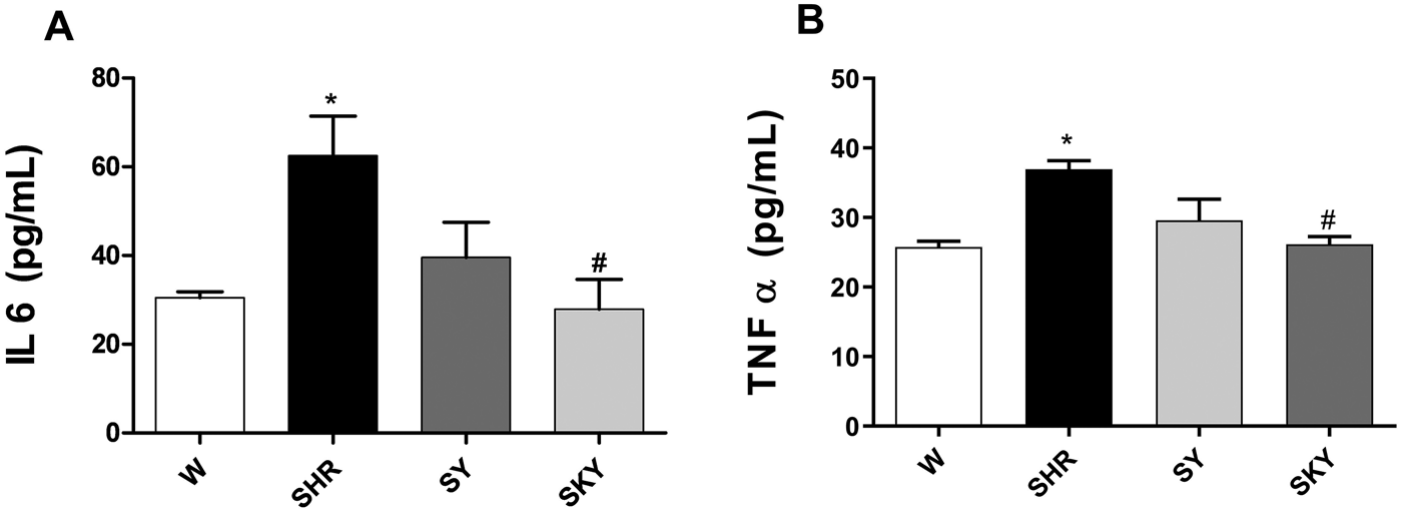
Analysis of inflammatory cytokines in the blood of animals. (A) Interleukin 6 (IL6); (B) tumour necrosis factor alpha (TNF α). Data are expressed as the mean ± S.E.M. Values were considered significant for ^*^ *p* < 0.05 vs. W. ^#^ *p* < 0.05 vs. SHR.

Phenolic compounds such as flavonoids protect against cardiovascular diseases, including hypertension and heart failure, by attenuating oxidative stress via Nrf2 activation and modulating additional pathways such as NF‐κB (Wu et al. 2022). Ferulic acid similarly protects against myocardial injury by improving cardiac function, reducing oxidative stress through Nrf2 activation, increasing GSH and SOD, and downregulating inflammatory signaling (Li et al. 2025).

Prebiotics, probiotics, and microbial metabolites improve gut microbiota homeostasis, maintain gut barrier integrity, and modulate immune responses (Liu et al. 2022). Gut dysbiosis during heart failure exacerbates cardiac dysfunction via TLR4/NF‐κB activation by endogenous LPS, increasing circulating IL‐6 and TNF‐α, and promoting myocardial inflammation and fibrosis (Zhang et al. 2025). Consistent with these findings, the synbiotic beverage altered gut microbiota composition, reduced oxidative stress markers (TBARS), decreased NF‐κB expression in cardiac tissue, and lowered systemic IL‐6 and TNF‐α.

Given the central role of oxidative stress in endothelial dysfunction and cardiac remodeling in hypertension (Ahmad et al. 2017; Alves et al. 2015; Ren 2007), and the link between chronic inflammation, angiotensin II signaling, and disease progression (Dalpiaz et al. 2013; Guzik et al. 2007), interventions modulating both oxidative and inflammatory pathways may provide advantages over approaches targeting blood pressure alone (Lin et al. 2013; Silva‐Cutini et al. 2019). Alterations in gut microbiota and metabolite production may influence circulating inflammatory mediators, contributing to oxidative stress and impaired cardiac function.

The LVM/BM ratio was higher in SHR and SY groups compared to W (*p* < 0.05). SKY treatment reduced LVM/BM relative to SHR and SY (*p* < 0.05), with no significant difference compared to W (*p* > 0.05; Figure 5). Final body weight did not differ significantly among the experimental groups (W: 305 ± 12 g; SHR: 289 ± 13 g; SY: 287 ± 11 g; SKU: 281 ± 10 g; *P* > 0.05).

**FIGURE 5:**
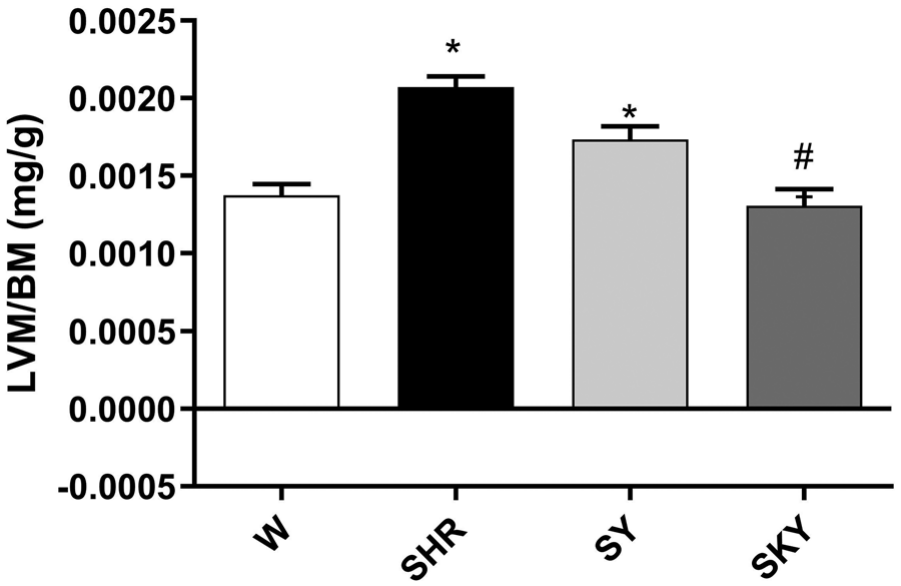
Left ventricular mass/body mass ratio (LVM/MC). Data are expressed as the mean ± S.E.M. The values were considered significant for ^*^ *p* < 0.05 vs. W; ^#^ *p* < 0.05 vs. SHR; ^+^ *p* < 0.05 vs. SY.

Diet‐derived microbial metabolites are key mediators of the gut–heart axis (Kondapalli et al. 2025; Snelson et al. 2025; Vashisht et al. 2026). The KY beverage attenuated cardiac hypertrophy in SHRs, supporting a cardioprotective effect and aligning with previous reports of structural cardiac benefits from fermented foods (Brasil et al. 2018; Silva‐Cutini et al. 2019; Lin et al. 2012). Although final body mass did not differ significantly among the experimental groups, cardiac mass normalization to tibia length was not performed, which should be considered when interpreting these findings. *Limosilactobacillus fermentum* enhanced the antihypertensive and antihypertrophic effects of hydrochlorothiazide in SHRs, likely via gut microbiota modulation, reduced oxidative stress, sympathetic suppression, and immune regulation (González‐Correa et al. 2025).

Hypertensive animals showed reduced fecal counts of *Bifidobacterium* sp. (Figure 6A) and *Lactobacillus* sp. (Figure 6B) compared to W (*p* < 0.05). KY treatment increased fecal counts of both species (*p* < 0.05) compared to SHR and SY, normalizing *Lactobacillus* growth relative to W. The Y beverage alone increased *Lactobacillus* counts compared to SHR.

**FIGURE 6:**
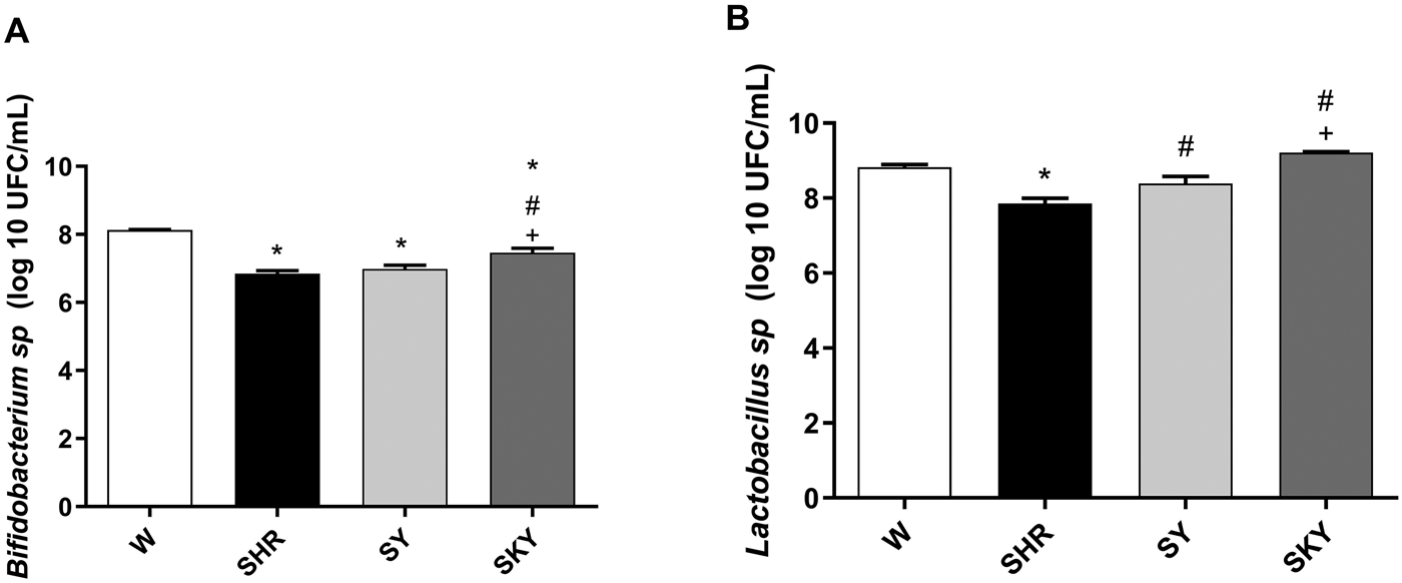
Evaluation of the growth of *Bifidobacterium* sp. (A) and *Lactobacillus* sp. (B) in the faeces of the experimental groups. Data are expressed as the mean ± S.E.M. The values were considered significant for ^*^ *p* < 0.05 vs. W; ^#^ *p* < 0.05 vs. SHR; ^+^ *p* < 0.05 vs. SY.

Diets and dietary compounds modulate gut microbiota composition and metabolite production (Abdolmaleky and Zhou 2024), contributing to antihypertensive effects (Tsai et al. 2013). Synbiotics improve microbiota diversity, nutrient absorption, and interactions between probiotics and prebiotics (Al‐Habsi et al. 2024). KY treatment increased fecal *Lactobacillus* spp. and *Bifidobacterium* spp. in SHRs, and *Lactobacillus* spp. in SY, consistent with reports linking fermented probiotic‐rich products to BP reductions and increased beneficial bacteria (Tsai et al. 2013; Robles‐Vera et al. 2020).

Gut microbiota‐derived metabolites, including SCFAs, mediate host–microbiota interactions that influence BP. SHR gut dysbiosis shows reduced acetate‐ and butyrate‐producing bacteria relative to WKY rats (Yang et al. 2015). Certain *Lactobacillus* strains reduce TMAO, a metabolite associated with elevated BP via oxidative stress and inflammation (Zhang et al. 2022; Ge et al. 2020; Yang et al. 2023).

Overall, the combined presence of bioactive phenolic compounds, enhanced antioxidant capacity, and probiotic microorganisms may contribute to cardiovascular homeostasis, improving BP, HR, and cardiac hypertrophy in SHRs, though individual contributions remain indistinguishable in this study (Habib et al. 2015; Dahal et al. 2020). SCFAs were not directly measured, but increased *Lactobacillus* and *Bifidobacterium* spp. likely enhanced SCFA production, potentially contributing to the observed effects (Wang et al. 2020; De Moura et al. 2023; Iqbal et al. 2025), which requires confirmation in future studies.

Some limitations of the present study should be acknowledged. First, the microbiological analysis was restricted to culture‐based quantification of selected cultivable bacterial populations, specifically *Bifidobacterium* spp. and *Lactobacillus* spp., and therefore does not provide a comprehensive characterization of the gut microbiota. Future studies employing 16S rRNA gene sequencing or other sequencing‐based approaches are warranted to determine whether KY induces broader changes in microbial composition and diversity. Second, WKY rats receiving Y or KY were not included in the experimental design. Consequently, although the interventions improved several alterations observed in SHR, the present study cannot determine whether these effects are specific to the hypertensive condition or would also occur in normotensive animals. Future studies including the corresponding intervention groups in normotensive animals will be important to distinguish hypertension‐related effects from more general physiological effects of the interventions.

## 4 Conclusion

In conclusion, the present study demonstrates that the kefir‐yacon fermented synbiotic beverage reduced blood pressure in SHR and modulated selected cultivable gut bacterial populations, accompanied by antioxidant and anti‐inflammatory effects associated with increased Nrf2 and reduced NF‐κB expression and lower systemic levels of inflammatory cytokines.

Overall, considering the limitations of the experimental design, these findings support the potential of plant‐based fermented synbiotic beverages as adjunct nutritional strategies for hypertension management. Further studies incorporating comprehensive gut microbiota analyses, additional experimental groups, and mechanistic approaches, followed by well‐designed clinical trials, are needed to confirm these findings, clarify the underlying mechanisms, and establish their translational relevance.

## Author Contributions

**Flavia de Souza Andrade Moraes**: conceptualization, project administration, formal analysis, writing – original draft, investigation, methodology. **Maressa de Carvalho Fragoso**: investigation, methodology, writing – review and editing, formal analysis, data curation. **Cristiane Lyrio da Silva**: investigation, methodology, formal analysis, data curation, validation. **Karynna Alcantara Dutra**: investigation, validation, methodology, formal analysis, data curation. **Lucas Gueller da Silva Gonçalves**: investigation, methodology, formal analysis, data curation. **Lucas Andrade Campelo**: investigation, methodology, formal analysis, data curation. **Antonio Ferreira de Melo Junior**: investigation, writing – review and editing, formal analysis, validation, methodology, resources, funding acquisition. **Nazare Souza Bissoli**: writing – review and editing, methodology, project administration, resources, funding acquisition, investigation. **Girlandia Alexandre Brasil Amorim**: funding acquisition, writing – review and editing, investigation, methodology, project administration, resources, supervision. **Ewelyne Miranda de Lima**: funding acquisition, investigation, writing – review and editing, project administration, supervision, resources. **Tadeu Uggere de Andrade**: conceptualization, investigation, funding acquisition, writing – review and editing, methodology, validation, formal analysis, project administration, supervision, data curation, resources.

## Conflicts of Interest

The authors declare no conflicts of interest.

## Supporting information




**Supplementary Materials**: jfds71598‐sup‐0001‐SuppMat.docx

## Data Availability

The data that support the findings of this study are available on request from the corresponding author.
