# Supplementary material for: Synbiotic Kefir‐Yacon Drink Ameliorates Hypertension by Modulating Antioxidant and Anti‐Inflammatory Pathways
Source: J Food Sci. 2026 Nov 9;91(11):e71598. doi: 10.1111/1750-3841.71598 (PMC13746308; doi:10.1111/1750-3841.71598)
Supplement: Supplementary file 1 [file JFDS-91-0-s001.docx]

**Supplementary material**

**Synbiotic Kefir-Yacon Drink Ameliorates Hypertension by Modulating Antioxidant and Anti-Inflammatory Pathways**

Flavia de Souza Andrade Moraesa, Maressa de Carvalho Fragosoa, Cristiane Lyrio da Silvaa, Karynna Alcantara Dutraa, Lucas Gueller da Silva Gonçalvesa, Lucas Andrade Campeloa, Antonio Ferreira de Melo Juniorb,c, Nazare Souza Bissolid, Girlandia Alexandre Brasil Amorima, Ewelyne Miranda de Limaa, Tadeu Uggere de Andradea*

**1. Western blotting procedure**

Western blotting was performed to assess total nuclear factor erythroid 2-related factor 2 (Nrf2), nuclear factor kappa B (NF-κB), superoxide dismutase (SOD), and catalase (CAT) protein expression in the left ventricle of the animals. Samples were homogenized in homogenization buffer containing 10 nM Tris-HCl, 5 nM EDTA, 1 nM PMSF, 1 nM NaVO₃, 0.5 nM DTT, 1% SDS, and a protease inhibitor cocktail (SigmaFast). Protein concentration was subsequently determined using the Bradford method (Bradford, 1976).

Proteins were separated by 10% SDS-polyacrylamide gel electrophoresis (SDS-PAGE) for 2.5 h at 100 V and subsequently transferred onto polyvinylidene fluoride (PVDF) membranes for 20 h using a wet protein transfer system. After transfer, the membranes were blocked with TBS-T containing 5% non-fat dry milk for 2.5 h.

The membranes were then incubated overnight at 4°C with appropriately diluted (1:1000) primary antibodies against catalase (CAT; Sigma, USA), SOD (Sigma, USA), Nrf2 (Sigma, USA), and NF-κB (Sigma, USA). After incubation, the membranes were washed with TBS-T and incubated with monoclonal anti-mouse IgG secondary antibodies (1:5000; Stressgen®, Victoria, Canada).

Protein bands were detected using a chromogenic substrate (NBT/BCIP Reagent Kit, Thermo Fisher Scientific, USA). An anti-glyceraldehyde-3-phosphate dehydrogenase (GAPDH) primary antibody (1:3000; Santa Cruz Biotechnology, Santa Cruz, CA, USA) was used as the loading control (Nascimento et al., 2016).

The molecular weight marker used in the present study was a colored protein standard (ColorBurst™ Electrophoresis Marker, C1992; Sigma-Aldrich). The marker was loaded in the first lane of each SDS-PAGE and transferred together with the samples from the gel onto the PVDF membrane.

**2. Original Western blot images**

The original Western blot images available from the experiments and used for densitometric analysis are provided in this Supplementary Material. The preserved images encompass the regions of the membranes containing the protein bands used for quantification. Full-length membrane images were not retained at the time of image acquisition.

For transparency, all original Western blot images available from the experiments are presented together with the corresponding 8-bit grayscale images used for densitometric analysis in ImageJ. Sample identification is provided for each lane, allowing correspondence between the original bands, grayscale images, and densitometric measurements.

Figures S1–S10 show the available original Western blot images and their corresponding grayscale images for Nrf2, NF-κB, SOD, CAT, and GAPDH.

**2.1. WB Images**

For each protein analyzed, two separate gels were performed. Each gel included samples from two animals from each experimental group: male Wistar rats treated with vehicle (W); male spontaneously hypertensive rats (SHRs) treated with vehicle (SHR); male SHRs treated with yacon (SY); and male SHRs treated with the fermented kefir-yacon beverage (SKY). Thus, each gel contained samples from eight animals, with a total of four animals per experimental group analyzed across the two gels.

**2.1.1. Superoxide dismutase (SOD)**

**Figure S1:** Western blot image of SOD – **Gel 1**. (A) Original Western blot image available from the experiment. (B) Corresponding 8-bit grayscale image used for densitometric analysis in ImageJ. The arrow indicates the SOD band selected for quantification. Samples from two animals from each experimental group (W1–W2, SHR1–SHR2, SY1–SY2, and SKY1–SKY2) were analyzed. The values shown below the images represent the densitometric intensity of each band, expressed in arbitrary units (a.u.).

**Figure S2:** Western blot image of SOD – **Gel 2**. (A) Original Western blot image available from the experiment. (B) Corresponding 8-bit grayscale image used for densitometric analysis in ImageJ. The arrow indicates the SOD band selected for quantification. Samples from two animals from each experimental group (W3–W4, SHR3–SHR4, SY3–SY4, and SKY3–SKY4) were analyzed. The values shown below the images represent the densitometric intensity of each band, expressed in arbitrary units (a.u.).

**2.1.2. Catalase (CAT)**

**Figure S3:** Western blot image of CAT – **Gel 1**. (A) Original Western blot image available from the experiment. (B) Corresponding 8-bit grayscale image used for densitometric analysis in ImageJ. The arrow indicates the CAT band selected for quantification. Samples from two animals from each experimental group (W1–W2, SHR1–SHR2, SY1–SY2, and SKY1–SKY2) were analyzed. The values shown below the images represent the densitometric intensity of each band, expressed in arbitrary units (a.u.).

**Figure S4:** Western blot image of CAT – **Gel 2**. (A) Original Western blot image available from the experiment. (B) Corresponding 8-bit grayscale image used for densitometric analysis in ImageJ. The arrow indicates the CAT band selected for quantification. Samples from two animals from each experimental group (W3–W4, SHR3–SHR4, SY3–SY4, and SKY3–SKY4) were analyzed. The values shown below the images represent the densitometric intensity of each band, expressed in arbitrary units (a.u.).

**2.1.3. Transcription factor Nrf2**

**Figure S5:** Western blot image of Nrf2 – **Gel 1**. (A) Original Western blot image available from the experiment. (B) Corresponding 8-bit grayscale image used for densitometric analysis in ImageJ. The arrow indicates the Nrf2 band selected for quantification. Samples from two animals from each experimental group (W1–W2, SHR1–SHR2, SY1–SY2, and SKY1–SKY2) were analyzed. The values shown below the images represent the densitometric intensity of each band, expressed in arbitrary units (a.u.).

**Figure S6:** Western blot image of Nrf2 – **Gel 2**. (A) Original Western blot image available from the experiment. (B) Corresponding 8-bit grayscale image used for densitometric analysis in ImageJ. The arrow indicates the Nrf2 band selected for quantification. Samples from two animals from each experimental group (W3–W4, SHR3–SHR4, SY3–SY4, and SKY3–SKY4) were analyzed. The values shown below the images represent the densitometric intensity of each band, expressed in arbitrary units (a.u.).

**2.1.4. Transcription factor NF-κB**

**Figure S7:** Western blot image of NF-κB – **Gel 1**. (A) Original Western blot image available from the experiment. (B) Corresponding 8-bit grayscale image used for densitometric analysis in ImageJ. The arrow indicates the NF-κB band selected for quantification. Samples from two animals from each experimental group (W1–W2, SHR1–SHR2, SY1–SY2, and SKY1–SKY2) were analyzed. The values shown below the images represent the densitometric intensity of each band, expressed in arbitrary units (a.u.).

**Figure S8:** Western blot image of NF-κB – **Gel 2**. (A) Original Western blot image available from the experiment. (B) Corresponding 8-bit grayscale image used for densitometric analysis in ImageJ. The arrow indicates the NF-κB band selected for quantification. Samples from two animals from each experimental group (W3–W4, SHR3–SHR4, SY3–SY4, and SKY3–SKY4) were analyzed. The values shown below the images represent the densitometric intensity of each band, expressed in arbitrary units (a.u.).

**2.1.5. Glyceraldehyde-3-phosphate dehydrogenase (GAPDH)**

**Figure S9:** Western blot image of GAPDH – **Gel 1**. (A) Original Western blot image available from the experiment. (B) Corresponding 8-bit grayscale image used for densitometric analysis in ImageJ. The arrow indicates the GAPDH band selected for quantification. Samples from two animals from each experimental group (W1–W2, SHR1–SHR2, SY1–SY2, and SKY1–SKY2) were analyzed. The values shown below the images represent the densitometric intensity of each band, expressed in arbitrary units (a.u.).

**Figure S10:** Western blot image of GAPDH – **Gel 2**. (A) Original Western blot image available from the experiment. (B) Corresponding 8-bit grayscale image used for densitometric analysis in ImageJ. The arrow indicates the GAPDH band selected for quantification. Samples from two animals from each experimental group (W3–W4, SHR3–SHR4, SY3–SY4, and SKY3–SKY4) were analyzed. The values shown below the images represent the densitometric intensity of each band, expressed in arbitrary units (a.u.).

**3. Densitometric analysis of Western blot bands using ImageJ**

Densitometric analysis of Western blot bands was performed using ImageJ software (National Institutes of Health, USA). The Western blot images were converted to 8-bit grayscale prior to analysis. Rectangular selections of identical dimensions were used to define the lanes corresponding to each sample. The lanes were analyzed using the **Analyze → Gels → Plot** Lanes function in ImageJ, which generates a densitometric profile in which each protein band is represented by a peak.

For each band of interest, the corresponding peak was identified in the densitometric profile, and the area under the peak was measured and used as the densitometric intensity of the respective protein band, expressed in arbitrary units (a.u.). The same procedure was applied to the corresponding GAPDH bands.

For each individual sample, the densitometric intensity of the target protein (Nrf2, NF-κB, SOD, or CAT) was normalized to the densitometric intensity of GAPDH from the corresponding animal, according to the following equation:

Normalized protein expression = densitometric intensity of target protein / densitometric intensity of GAPDH

Thus, an individual target protein/GAPDH ratio was obtained for each animal. The mean normalized value of the control group (W) was subsequently set to 1 (equivalent to 100%), and the normalized values of each individual sample were expressed relative to the mean of the control group according to the following equation:

Relative protein expression = (target protein/GAPDH ratio of the individual sample) / (mean target protein/GAPDH ratio of the W group)

Accordingly, the mean value of the control group corresponds to 1, and values above or below 1 indicate higher or lower relative protein expression, respectively, compared with the control group.

The resulting relative expression values were used for statistical analysis and graphical presentation of Nrf2, NF-κB, SOD, and CAT protein expression.

**4. References**

Bradford, M. M. (1976). A rapid and sensitive method for the quantitation of microgram quantities of protein utilizing the principle of protein-dye binding. *Analytical Biochemistry*, *72*(1–2), 248–254. <https://doi.org/10.1016/0003-2697(76)90527-3>

Nascimento, A. M. do, Lima, E. M. de, Brasil, G. A., Caliman, I. F., Silva, J. F. da, Lemos, V. S., … Bissoli, N. S. (2016). Serca2a and Na + /Ca 2 + exchanger are involved in left ventricular function following cardiac remodelling of female rats treated with anabolic androgenic steroid. *Toxicology and Applied Pharmacology*, *301*, 22–30. <https://doi.org/10.1016/j.taap.2016.04.001>
